# Cell Force-Driven Basement Membrane Disruption Fuels EGF- and Stiffness-Induced Invasive Cell Dissemination from Benign Breast Gland Acini

**DOI:** 10.3390/ijms22083962

**Published:** 2021-04-12

**Authors:** Aljona Gaiko-Shcherbak, Julian Eschenbruch, Nils M. Kronenberg, Michael Teske, Benjamin Wolters, Ronald Springer, Malte C. Gather, Rudolf Merkel, Bernd Hoffmann, Erik Noetzel

**Affiliations:** 1 Institute of Biological Information Processing 2 (IBI-2): Mechanobiology, Forschungszentrum Jülich, 52425 Jülich, Germany; agaiko-shcherbak@clinipace.com (A.G.-S.); j.eschenbruch@fz-juelich.de (J.E.); michael.teske@uzh.ch (M.T.); benjamin.wolters@evotec.com (B.W.); r.springer@fz-juelich.de (R.S.); r.merkel@fz-juelich.de (R.M.); b.hoffmann@fz-juelich.de (B.H.); 2 SUPA, School of Physics and Astronomy, University of St Andrews, St Andrews KY16 9SS, UK; nils.kronenberg@uni-koeln.de (N.M.K.); mcg6@st-andrews.ac.uk (M.C.G.); 3 Humboldt Centre for Nano- and Biophotonics, Department of Chemistry, Universität zu Köln, 50939 Köln, Germany

**Keywords:** basement membrane, mechanobiology, mechanically driven cancer progression, mechanosensing, neoplasm invasion, breast cancer invasion, cell force, mechanosensory transduction, filopodia

## Abstract

Local basement membrane (BM) disruption marks the initial step of breast cancer invasion. The activation mechanisms of force-driven BM-weakening remain elusive. We studied the mechanical response of MCF10A-derived human breast cell acini with BMs of tuneable maturation to physical and soluble tumour-like extracellular matrix (ECM) cues. Traction force microscopy (TFM) and elastic resonator interference stress microscopy (ERISM) were used to quantify pro-invasive BM stress and protrusive forces. Substrate stiffening and mechanically impaired BM scaffolds induced the invasive transition of benign acini synergistically. Robust BM scaffolds attenuated this invasive response. Additional oncogenic EGFR activation compromised the BMs’ barrier function, fuelling invasion speed and incidence. Mechanistically, EGFR-PI3-Kinase downstream signalling modulated both MMP- and force-driven BM-weakening processes. We show that breast acini form non-proteolytic and BM-piercing filopodia for continuous matrix mechanosensation, which significantly push and pull on the BM and ECM under pro-invasive conditions. Invasion-triggered acini further shear and compress their BM by contractility-based stresses that were significantly increased (3.7-fold) compared to non-invasive conditions. Overall, the highest amplitudes of protrusive and contractile forces accompanied the highest invasiveness. This work provides a mechanistic concept for tumour ECM-induced mechanically misbalanced breast glands fuelling force-driven BM disruption. Finally, this could facilitate early cell dissemination from pre-invasive lesions to metastasize eventually.

## 1. Introduction

Malignant transformation of human breast gland tissue is regulated at the level of reciprocity between cells and their microenvironment [1]. Mechanical tissue homeostasis is a crucial gatekeeper of tumour progression. In this context, chronically misbalanced tensile tissue stresses caused by pre-malignant epithelial cells could accelerate cell force generation and invasive outgrowth [2]. Growth factors and extracellular matrix (ECM) proteolysis could further modulate this tumorous progression [3,4]. However, the cellular mechanisms behind the complex feedback loops of physical and biochemical ECM cues and cellular response could trigger cell invasion are still under debate [5,6,7]. Basement membranes (BMs) are dense and highly cross-linked ECM scaffolds and essential cell platforms regulating embryogenesis, wound healing, and tissue homeostasis [6]. During normal epithelial tissue development and cellular immune surveillance, BMs provide tissue compartmentation by tightly regulating cell transmigration [8]. Accordingly, one hallmark to discriminate benign from malignant invasive breast carcinomas is the lack of a BM barrier [9]. During cancer progression, invasive cells disseminate from such expanding and infiltrating tumour masses to form distant metastasis causing high mortality rates of patients [10]. Controversially, a growing body of evidence reveals that a large proportion of breast cancer patients (44%) with non-invasive *ductal carcinoma* in situ *(DCIS)* suffer from putative metastasizing circulating tumour cells (CTCs) [11]. These CTCs could relate to the substantial amount of distant metastases that originate from low-grade rather than invasive high-grade primary tumours [12]. Together this suggests that pre-invasive cells can disseminate very early from pathologically non-invasive *carcinoma* in situ *(CIS)* by activation of specific mechanisms for local BM invasion.

The investigation of cellular BM transmigration mechanisms under normal and tumorous tissue conditions remains challenging due to the experimental limitations of capturing cell–BM interactions in vivo [6]. Nevertheless, landmark experiments used BM explants from rat peritoneum to reveal the crucial role of cell membrane-bound matrix metalloproteases (MT-MMPs) for breast cell invasion [4]. Ground-breaking studies demonstrated the pro-invasive function of ECM stiffness and MMP-dependent ECM proteolysis [13]. ECM rigidity is a fundamental regulator of cell contractility, cell adhesion, migration, proliferation, and gene expression contributing to breast cancer development, progression and invasion [14,15]. Cells sense and transduce ECM stiffness cues via force transmitting focal adhesions (FAs) coupled with contractile actomyosin filaments [16]. At the single cell level high cellular traction forces have been suggested to drive cell adhesion and migration in breast cancer progression [17].

We investigated the still unknown regulation of force-driven BM disruption modes activated by non-malignant breast cells in tumorous microenvironments. We used benign MCF10A breast acini covered with BM scaffolds of low and high mechanical strengths [18,19,20] to explore the BMs’ capacity to compensate cellular mechanical stresses and attenuate cell invasion. By adopting traction force microscopy (TFM) [21] to a newly designed in vitro BM invasion assay, we measured the impact of chronically high amplitudes of physical BM stresses and mechanical cell–substrate interactions on invasion speed and incidence. We used tumour-like substrate stiffening and oncogenic EGF stimulation to modulate the amplitudes of mechanical BM stresses to potentiate the invasive transition of non-malignant cells. Elastic resonator interference stress microscopy (ERISM) [22] discovered invasion-related, oscillatory and BM-stressing filopodia that benign breast acini use for trans-BM mechanosensation. Together, our work provides a novel perspective on local BM disruption at sites of pre-invasive breast cancer lesions within benign tumour cells activating an early route of metastasis.

## 2. Results

### 2.1. Substrate Stiffening, Impaired BM Mechanics and Aberrant EGF Induce Invasion Synergistically

**Figure 1:**
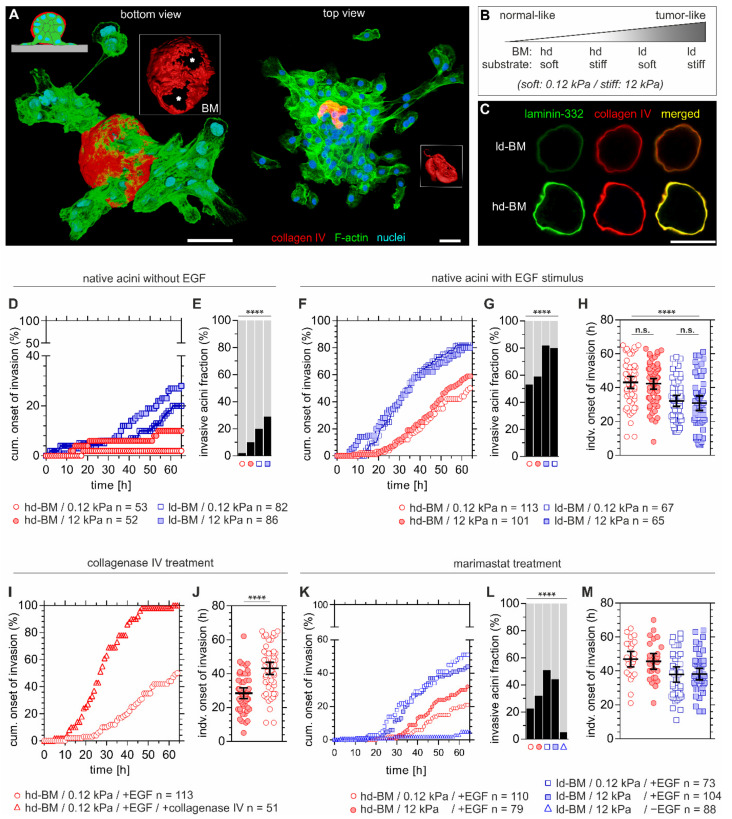
Basement membrane (BM) mechanics counteract tumour extracellular matrix (ECM)-induced invasion. (**A**) 3D reconstructed confocal images showing a local basement membrane (BM) disruption and cell invasion event (left acinus) on a 12 kPa PDMS silicone rubber substrate. Left: Local BM disruption foci (asterisks) and collective cell transmigration are present at the cell–BM–substrate interface. Right: Massive cell dissemination and a collapsed, cell-free BM hull (red), indicating the late invasion phase. (**B**) Experimental conditions to resemble the shift from physiological to malignant breast tissue states by altering BM development and substrate compliance. (**C**) MCF10A breast acini-derived BMs with progressive developmental stages. Micrographs are illustrating ld-BM and hd-BM structures with representative increasing deposition of BM-specific marker proteins type IV collagen and laminin-332. (**D**) Cumulative events of BM transmigration onset over time and (**E**) fraction of invasive acini after 65 h and different BM states and substrate compliance levels. (**F**) Cumulative BM transmigration onset, (**G**) fraction of invasive acini after 65 h and (**H**) and individual invasion times for EGF-pretreated acini, comparing again different BM states and substrate compliance levels. (**I**) Cumulative BM transmigration after type IV collagenase and EGF pretreatment and (**J**) corresponding individual times of invasion for hd-BM acini soft substrate. (**K**) Cumulative BM transmigration, (**L**) fraction of invasive acini after 65 h and (**M**) corresponding times of invasion for individual acini after pretreatment with MMP inhibitor marimastat, again for different BM states, substrate compliance levels and ±EGF. n: sample size of at least three independent experiments (see Table S1 for exact invasive fraction values and mean invasion timepoints). Scatter plots (**H**,**J**,**M**): bars: mean with 95% confidence interval (CI). Scale bars: 50 µm; n.s.: *p* ≥ 0.05; ****: *p* < 0.0001).

We found that placing non-invasive MCF10A breast acini onto stiff elastomeric substrates (12 kPa) triggers local BM disruption and cell dissemination (Figure 1A). To investigate the impact of healthy and pathologic mechanical tissue states on cell invasion, we cultured BM-covered breast acini on elastomeric substrates with rigidity of normal/soft (0.12 kPa) and tumour-like/stiffened (12 kPa) breast gland tissue [23] (Figure 1B). To determine the pro-invasive effect of tumour-related BM thinning/weakening, we analysed acini featuring BMs of different thicknesses and mechanical strengths. These sample types are defined as acini with low developed BM scaffolds (ld-BM acini, 10 days cultivation time) and highly developed BMs (hd-BM acini, 21 days cultivation time) (Figure 1B,C).

Hd-BM acini seeded on soft substrates (12 kPa) showed only neglectable invasiveness (2%). Accordingly, intermediate tissue states resembled by either a stiff substrate or an ld-BM scaffold resulted in moderate invasive outcomes of 10% and 20%, respectively. In contrast, the highest invasion speed and incidence correlated with substrate stiffening and ld-BMs (Figure 1D). Strikingly, there was a 5.6-fold increased incidence (28%) for this most progressed tumour condition (12 kPa/ld-BM) compared to the hd-BM/soft substrate group (*p* < 0.0001, Figure 1E). These results indicated a synergistic action of both substrate stiffening and low mechanical BM strength on local BM disruption and cell transmigration.

Interestingly, about two-thirds of non-malignant breast acini remained in non-invasive states. We next tested the impact of oncogenic growth factor signalling on BM disruption. Pretreatment with EGF drastically increased the speed and incidence of invasion in all sample groups, up to 82% (Figure 1F,G), and overwrote the previously observed gradual substrate-stiffening effect. Approximated timepoints of BM disruption on soft/stiff substrates underlined this EGF effect (Figure 1H). By contrast, the anti-invasive effect of mechanically robust BM scaffolds remained evident since hd-BM acini showed remarkably lower incidences (53% and 59%) compared to the most invasive ld-BM acini groups (80% and 82%) (Figure 1G). In line with this, transmigration was substantially delayed by about 11 h (hd-BM group means: 42.3 and 43.0 h and ld-BM group means: 30.9 and 32.2 h, *p* < 0.0001) (Figure 1H).

### 2.2. Benign Breast Acini Use a Non-MMP-Dependent Mode of BM-Weakening

As the BM developmental state strongly influenced the invasion outcome, we next targeted the mechanical BM integrity. Indeed, enzymatic-weakening of the structure forming the collagen IV network [18] in originally low-invasive hd-BM samples resulted in 100% incidence and substantially earlier BM transmigration onset (14.6 h, *p* < 0.0001) (Figure 1I,J). To test whether BM proteolysis contributed to acinar invasion, samples were also treated with the broad-spectrum MMP inhibitor marimastat. The invasion incidence of marimastat treated acini was clearly lower (ld-BM groups: 23% and 44%, Figure 1K,L) than for native acini. Moreover, compared to the untreated groups, marimastat delayed BM transmigration on average by 4 h in hd-BM (means: 46 and 47 h) and 6 h in ld-BM acini (means: 38 h) (Figure 1M).

Without oncogenic EGF stimulation the effect of MMP inhibition was even more evident. A nearly complete blockade was evident, even for highly invasive acini (down to 5%, ld-BM acini, stiff substrate, Figure 1L). Without MMP inhibition, such low transmigration was only found for the most physiological group (hd-BM, soft substrate, Figure 1E). The significant fraction of breast acini that remained highly invasive in the presence of marimastat and EGF indicates an at least partially MMP-independent cellular mechanism of BM disruption.

### 2.3. Benign Acini Exert the Highest Mechanical BM Stresses in Tumorous ECM Conditions

**Figure 2:**
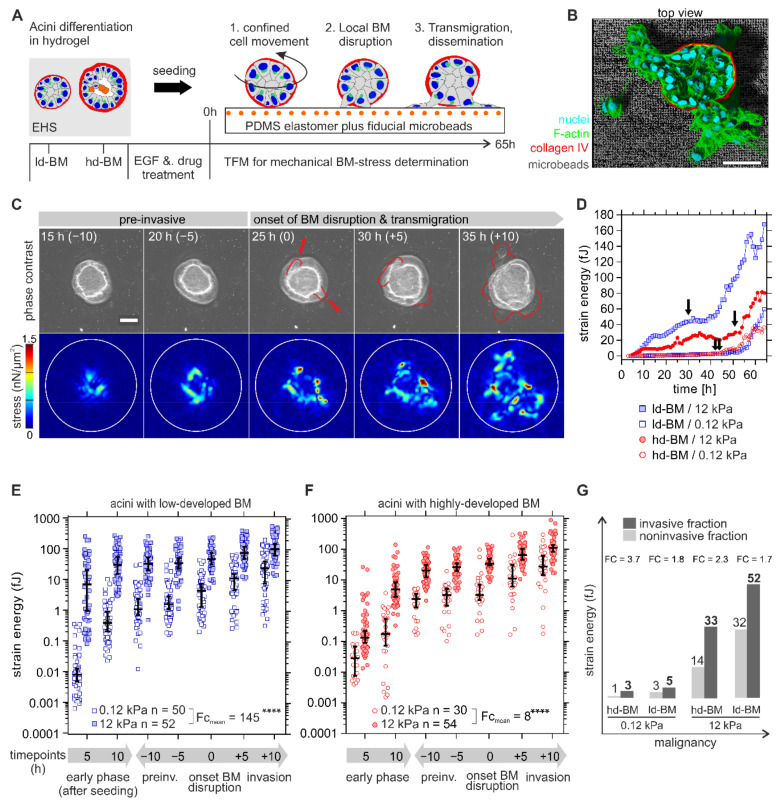
Mechanical BM stress exertion by benign breast acini. (**A**) Scheme of the designed traction force microscopy (TFM) assay to determine invasion-associated physical BM stress. The experimental workflow illustrates the general sequence of events in response to BM developmental stage, substrate stiffening, and EGF stimulation on cell invasion. (**B**) 3D rendered image shows an invasive hd-BM acinus on a stiff TFM substrate (12 kPa PDMS rubber) and incorporated fluorescent fiducial microbeads. (**C**) Representative TFM image sequence showing tangential mechanical stress exerted at the BM–substrate interface by an ld-BM acinus (+EGF) on 12 kPa substrate at timepoints before (pre-invasive), at BM disruption and during cell transmigration. Upper row: phase-contrast images illustrate the first appearance of protrusive cell bodies (red outlines), marking the onset of BM disruption and transmigration. Timepoints (in brackets) correspond to the dichotomized *x*-axis used in (**E**–**G**). Lower row: matching stress maps (nN/µm^2^) used for strain energy calculation. White outlines: ROI for strain energy calculation. (**D**) Representative strain energy regimes of individual acini (continuous TFM data, arrows indicate BM disruption events). (**E**,**F**) Overall comparison of detected strain energies (BM stress) according to altered BM and ECM conditions. *X*-axis: the first 5 and 10 h after acini seeding define the *early phase*. Other timepoints are normalized to the individual *onset of BM disruption* (=0 h) and dichotomized into comparable *pre-* (“- hours”) and *post-invasive* (“+ hours”) *phases* (see Table S2 for exact median strain energy values plotted in (**E**,**F**)). (**G**) The graphic illustrates the relationship between BM stress and malignancy. A grouped comparison of median strain energies at matching timepoints in non-invasive (at measurement intervals of 20, 30, 40, 50, 60 and 65 h) and invasive fractions (at timepoints of BM invasion) depending on ECM conditions. FC: calculated from median values. Scatter bars: median with 95% CI. n: the invasive sample size of at least three independent experiments. Mean fold change (FC_mean_): the ratio of all median strain energies averaged over all timepoints. Scale bars: 50 µm; ****: *p* < 0.0001).

Having found the first piece of evidence for an MMP-independent invasion, we next tested by TFM for cell-derived mechanical BM stresses that contribute to BM invasion. Long-term observation of breast acini seeded onto elastomeric substrates showed three distinct phases: (1) confined cell movement within the intact BM shell, (2) local BM disruption, and finally (3) cell transmigration and dissemination (Figure 2A,B). The representative image sequence in Figure 2C illustrates the mechanical interaction of an ld-BM acinus with a stiff substrate during the aforementioned phases. Here, first protrusive cells emerged underneath the acinar body at 25 h and thus marked the timepoint of BM invasion onset with subsequent cell migration into the microenvironment (≥30 h) (Figure 2C, red arrows, upper row). A steady increase in traction before (pre-) and at the onset of BM disruption, as well as during cell transmigration, was recorded (Figure 2C, lower row).

An essential first finding was the significant and dynamic stress pattern at the BM–substrate interface that occurred in the pre-invasive phase (see also Movies S1 and S2 for complete image sequences for ld-BM and hd-BM acini). For further analysis, strain energy was calculated as a robust and scalar measure for cellular traction force [24,25] (for detailed information, see also Section 4.7). The terms cell force and BM stress are equivalently used for the measured strain energy. The data for a representative set of acini in Figure 2C show that both ld- and hd-BM acini exerted considerably higher pre-invasive strain energies (median 25 and 20 fJ) on stiff as opposed to soft substrates (median 2.4 and 2.7 fJ). After local BM disruption, a second sharp increase in energy indicated cell transmigration and dissemination (cf. Figure 2D, arrows and Figure 2C, 25–35 h).

Statistical evaluation of n = 186 acini required dichotomization of the continuous and naturally scattered data sets into comparable pre- and postinvasion phases (Figure 2E,F). All analysed samples were distinguishable above the median baseline of technical controls (FC ≥ 4, see also Figure S1) This approach confirmed the presence of significant strain energies in the early pre-invasive phase after acini seeding (first 5 to 10 h) that further increased during the following pre-invasive phase (indicated as −10 to −5 h) before the onset of actual BM disruption (at 0 h). Finally, strain energies peaked for all sample groups within the invasive phase (+5 to +10 h after BM disruption) when extensive cell migration took place (Figure 2E,F). What stood out was the fact that BM stresses were overall smallest on normal-like/soft substrates for both ld-BM and hd-BM breast acini (range: 0.01–3.25 fJ, cf. Figure 2E,F).

Figure 2E compares the strain energies induced by ld-BM acini on soft and stiff substrates. It turned out that the most invasive acini group also generated the highest BM stress (ld-BM acini on stiff substrate; median_all timepoints_: 37 fJ, range: 7.5–105 fJ). This substrate-stiffening effect was most pronounced during the early pre-invasive phase (first 5 to 10 h). Compared to soft substrates, the same acini group showed massively enhanced mean strain energies of approx. 860-fold and 80-fold. However, substrate stiffening resulted in an overall increase in strain in ld-BM acini (approx. FC_mean_ = 140). Figure 2F shows the force values of hd-BM acini on soft and stiff substrates; overall, hd-BM acini showed 10-fold smaller strain values than ld-BM acini even on stiff substrates.

Nevertheless, hd-BM acini interacting with stiff substrates exerted the second largest strain energies (median_all timepoints_: 26 fJ, range: 0.13–111 fJ) and concordantly correlated with the second highest invasive outcome. As demonstrated for ld-BM acini, a similar, though less pronounced substrate stiffness-induced stress generation was also evident for hd-BM acini (approx. FC_mean_ = 8). These data demonstrate that substrate stiffness and BM mechanics significantly affected cell force generation in the very early phase of invasive transition.

To functionally link increased BM stress to invasiveness, we compared the force values of invasive acini at onset of BM disruption with matching timepoints for non-invasive samples. This approach revealed that all invasive acini showed distinguishably higher BM stress levels than their non-invasive counterparts (FC = 1.7 to FC = 3.7) (Figure 2G). This plot further verified the pro-invasive impact of BM stress amplitudes at timepoints of BM disruption when increased by progressive tumour conditions (Figure 2G).

EGF is a potent inducer of cell division and substantially fuelled the invasive transition (cf. Figure 1E,G). We compared the proliferation rates within acinar bodies in normal and tumour conditions to test for the contribution of growing expanding cells to the observed mechanical BM stress. EGF-induced proliferation was highest in the most invasive acini groups (ld-BM soft/stiff: 26 and 28%) compared to the respective untreated controls (<15%). In contrast, low-invasive hd-BM groups showed only basal proliferation rates (<2%) and appeared to be relatively unaffected by additional EGF stimulation (<5%) (Figure S2). Of note, the EGF-induced proliferation matched the highest amplitudes of pre-invasive BM stresses measured for the most invasive acini groups.

Altogether our findings indicate that environmental strain energies caused by acinar forces correlate in a continuous way with tumour progressive breast tissue states and invasive outcome. In less technical words, the physical BM stress transmitted by cell forces increases significantly with tumour progression and invasiveness.

### 2.4. Benign Acini Use Non-Proteolytic Filopodia for Trans-BM Mechanosensation

**Figure 3:**
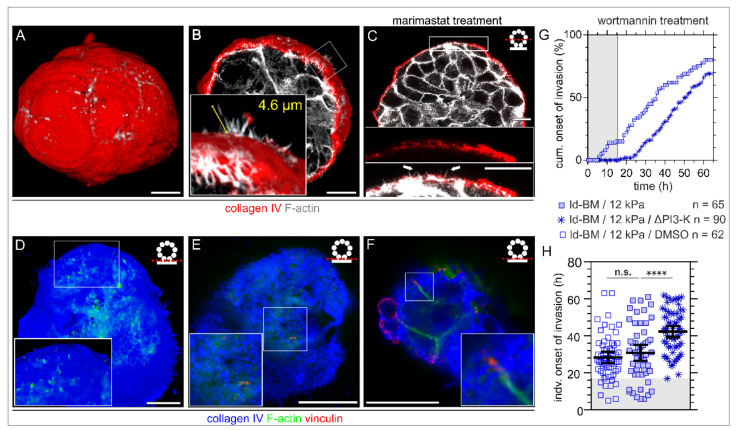
Formation of BM traversing actin protrusions and the impact of PI3-kinase inhibition on cell invasion. MCF10A acini were fixed and IF-stained. (**A**) 3D reconstructed confocal image stack demonstrating BM (collagen type IV, red) penetrating actin-based protrusions (F-actin, white) formed in physiological conditions within EHS matrix (fully matured hd-BM acinus after 31 days). (**B**) 3D cross-section through an hd-BM acinus with BM (red)-piercing F actin-rich protrusions (white) for proinvasive conditions (+EGF, 24 h on glass) including length measurement (yellow). (**C**) 2D cross-section of a marimastat treated ld-BM acinus (+EGF, 24 h on glass), indicating the formation of actin-rich protrusions (white arrow heads) piercing collagen type IV pores of the BM. (**D**) Rendered 3D image stack shows the acini–BM–substrate interface before BM transmigration started (hd-BM, 48 h on glass). Protrusive microspikes (F-actin, green) are visible in between the porous BM scaffold (collagen type IV, blue) reaching the underlying substrate. (**E**) Detailed view on a preinvasive hd-BM acinus adhered to a 3 kPa substrate demonstrates the colocalization of F-actin spots (green) and vinculin patches (red) within collagen pores (blue) at the BM–substrate interface. (**F**) The BM–substrate interface of an hd-BM acinus at the BM transmigration onset timepoint shows F-actin bundles with vinculin patches adjacent to the substrate plane (rigid glass). (**G**) The cumulative onset of invasion in ld-BM breast acini on stiff (12 kPa) substrate (+EGF) pretreated with the specific PI3-K inhibitor Wortmannin (ΔPI3-K). (**H**) Scatter plot showing the shift of the individual invasion onset timepoints of treated and untreated control groups. Bars: mean with 95% CI. n: analysed samples of at least three independent experiments. Grey plotted areas: complete invasion block for the first 17 h in wortmannin treated acini. Scale bars: 10 µm; n.s.: *p* ≥ 0.05; ****: *p* < 0.0001).

The induction of significant BM stress at early pre-invasive phases raised the question of how breast acini cells interact with the BM–substrate interface to transduce substrate stiffness information into cell forces. Immunofluorescence-based analysis revealed F-actin-rich cell protrusions penetrating the intact BM scaffold of non-invasive breast acini. These finger-shaped protrusions were randomly distributed over the acinar body both on hd-BM (Figure 3A) and on ld-BM acini (Figure S3A), interacting with a 3D EHS matrix. Notably, these lateral protrusions were also formed by invasion-triggered acini. Figure 3B shows a protrusion hot spot on an hd-BM acinus treated with EGF on a tumour-like substrate. These filopodial protrusions grow from short actin-spikes and gained lengths of >4 µm. Their small diameters (<1 µm) were in the range of the pore sizes of the collagen IV meshwork (Figure 3B). The MMP inhibitor treatment demonstrated that those actin spikes pierce the BM without the proteolytic activity of membrane-bound MMPs inhibited by marimastat (Figure 3C). Detailed investigation of the BM–substrate interface before and at the onset of BM disruption further revealed BM-piercing F-actin spikes independent of substrate stiffness (Figure 3D, 3 kPa; and Figure 3F, glass). These vertically protruding microspikes were frequently colocalized with characteristic vinculin patches, indicating force transmitting cell–matrix adhesion complexes (see zoomed insets in Figure 3E,F). For a complementary IF series, see Figure S3.

To functionally link these ECM sensory filopodia with the BM invasion process, we targeted the activation of the Phosphoinositide 3-kinase (PI3-K), a central signalling hub for the formation of filopodia and invadopodia. Intriguingly, wortmannin treatment completely blocked transmigration of originally highly invasive ld-BM acini for approx. 17 h (Figure 3G, grey box) and substantially delayed (+13 h) the mean invasion onset to 42 h (Figure 3H), a value found before only for low invasive hd-BM acini (43 h, Figure 1H, left column). However, the overall invasion incidence was less affected, which might be technically related to an activity loss of the drug after 24 h restoring the acini’s invasive response. A low dose of wortmannin and a short incubation time were ensured to minimize unspecific side effects.

### 2.5. Invasion-Triggered Breast Acini Push and Pull Their Microenvironment

**Figure 4:**
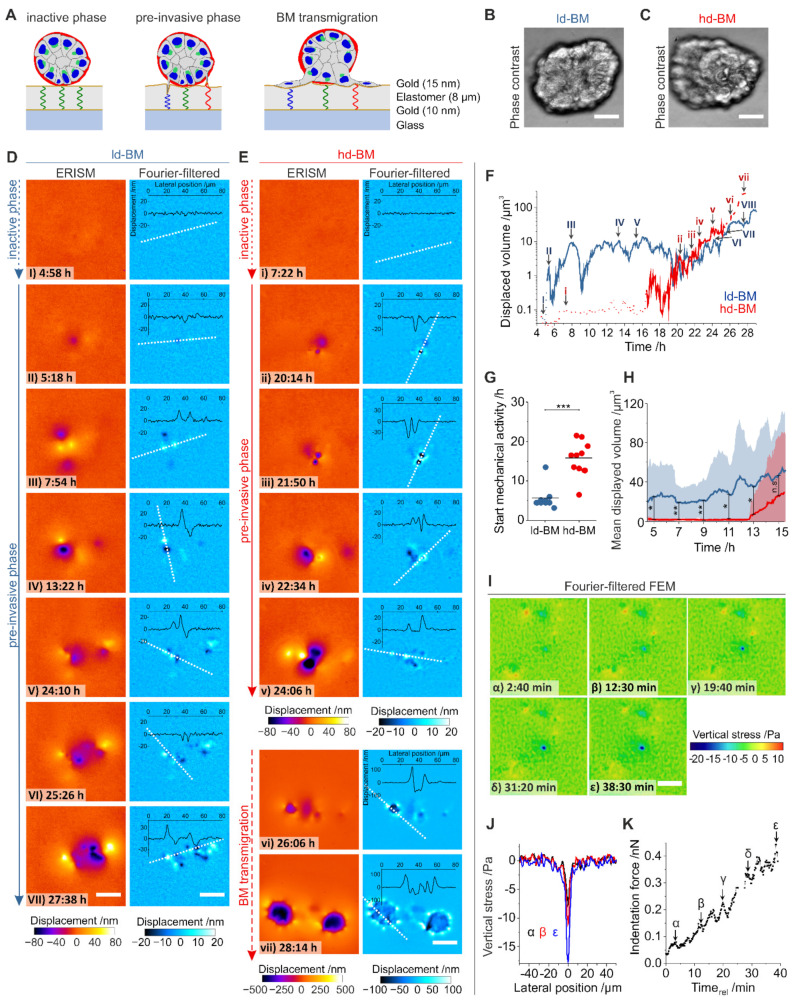
Measurement of mechanically active protrusions of pre- and post-invasive breast acini. (**A**) Schematic illustration of the elastomer resonance interference spectroscopy microscopy (ERISM) measurement. An acinus is seeded on an elastic and soft ERISM chip (apparent stiffness: 3 kPa, functionalized with type IV collagen). During the pre-invasive phase, cell protrusion forces deform the ERISM chip locally, leading to local changes to the resonance wavelengths of the ERISM chip, which are detected optically. After BM transmigration, cells adhere to the chip surface exerting contractile force. (**B**) Phase-contrast image of a representative ld-BM acinus and (**C**) of a hd-BM acinus. (**D**) ERISM displacement maps (left column) and Fourier-filtered ERISM displacement maps (right column) for the ld-BM acinus shown in (**B**) at different timepoints. Insets show lateral profiles along the white dotted lines. (**E**) ERISM displacement maps (left column) and Fourier-filtered ERISM displacement maps (right column) for the hd-BM acinus shown in (**C**) at different timepoints. Insets show lateral profiles along the white, dotted lines. (**F**) Total substrate volume displaced by mechanical activity of the ld-BM acinus in (**D**) (blue line) and the hd-BM acinus in (**E**) (red line) as a function of the time after seeding. Dotted, solid and dashed lines indicate the initial mechanically inactive phase, the pre-invasive phase and the phase after the onset of BM transmigration. Uppercase Latin numbers indicate the timepoints of the displacement maps for the ld-BM acinus shown in (**D**). Lowercase Latin numbers indicate the timepoints of displacement maps for the hd-BM acinus shown in (**E**). Recording: 4 min/frame over 4.5–29.0 h after seeding. (**G**) Comparison of the time from seeding to start of pre-invasive mechanical activity in ld-BM (blue; n = 9) and hd-BM (red; n = 10) acini. (**H**) Mean substrate volume displaced by ld-BM (blue line; n = 9) and hd-BM acini (red line; n = 10) as a function of time after seeding. Shaded regions indicate root-mean-square variation. (**I**) Maps of vertical stress showing the advance of a single cell protrusion of an ld-BM acinus. Recording: 1 frame every 10 sec over 45 min. (**J**) Lateral profiles through the point of maximum stress in (**I**) at the three different timepoints. (**K**) Temporal evolution of the indentation force by the single cellular protrusion shown in (**I**). Force calculated by spatial integration of the stress over the area of the protrusion—i.e., where stress ≤ 3 kPa. All scale bars: 25 µm; n.s.: *p* > 0.05; *: *p* ≤ 0.05; **: *p* ≤ 0.01; ***: *p* ≤ 0.001.

Based on the finding that acinar protrusions contact the underlying ECM through the intact BM barrier, we aimed to functionally link such protrusion activity with mechanical substrate deformation/displacement. We used ERISM, a recently developed method for vertical stress mapping that is based on optical interference [22]. ERISM resolved any substrate deformations caused by BM-piercing protrusions and later during BM transmigration and computed the corresponding mechanical stresses (Figure 4A). Proinvasive conditions (+EGF and stiff substrate) were chosen to measure the mechanical activity of protrusions formed by ld-BM and hd-BM acini (Figure 4B,C).

Figure 4D,E show representative displacement maps from a 24 h time-lapse ERISM measurement for different timepoints starting 4.5 h after seeding for the ld-BM and an hd-BM acinus, respectively (see Movie S3 and S4 for complete image sequences). At the start of the measurement, substrate displacements were barely visible, indicating that the acini show insignificant mechanical activity (inactive phase, Figure 4(DI,Ei)). However, at 5.3 and 16.5 h after seeding, respectively, the ld-BM and hd-BM acini started to locally deform the substrate by pulling and pushing its surface, indicating the start of the pre-invasive phase (Figure 4(DII,Eii)). To quantify the force each acinus exerted, we calculated the total volume by which they indented into the substrate for all ERISM maps of the time-lapse series (Figure 4F). This showed that the ld-BM acinus exerted a strong and oscillatory force over most of the measurement, while the hd-BM acinus showed negligible force until the onset point at 16.5 h, but then also delivered increasing and highly dynamic force.

Investigation of several acini confirmed the earlier onset of pre-invasive pushing and pulling for ld-BM acini (mean ± SEM; ld-BM: 5.7 h ± 1.0 h, n = 9 hd-BM: 15.8 h ± 1.4 h, n = 10; *p* = 0.001, Figure 4G; for complete datasets see Figure S4) and showed a significantly increased mean displaced volume during the first 15 h after seeding for ld-BM acini (on average by 60-fold, Figure 4H). However, after the onset of mechanical activity in both ld-BM and hd-BM acini, the difference in the mean substrate indentation was no longer significant (ld-BM: 14.7 µm^3^ ± 1.4 µm^3^, n = 3; hd-BM: 22.2 µm^3^ ± 7.7 µm^3^, n = 7, *p* = 0.55; Figure S5).

Spatial Fourier filtering of ERISM maps can remove any broad deformation features and thus uncover subtle and locally confined substrate pulling and pushing sites [22,26]. Applying this method to acini data revealed features underneath the acinar bodies that suggested the involvement of the protrusions described in Figure 3 (Figure 4D,E). Pulling and pushing sites occurred both spatially adjoined and clearly separated (>50 µm), with the latter suggesting several mechanically active cells within one acinus. Pre-invasive pushing and pulling sites were transiently formed and released, causing rapidly changing displacement patterns (Movies S3 and S4) and explaining the strong oscillation of the total exerted force in Figure 4F. The number of pushing and pulling sites increased during the pre-invasive phase, causing an overall increase in displaced substrate volume with time.

To further investigate stress exertion of individual protrusions, a high framerate ERISM measurement of a single protrusion was converted into maps of vertical stress using finite element modelling (FEM; Figure 4I; complete data set, Movie S5). The stress exertion was spatially highly confined (<4–5 µm; Figure 4J) and the protrusive force increased with a tooth-shaped oscillatory pattern (period: 4.0 min; Figure 4K), reaching a maximum of 0.4 nN at the end of the measurement. Opposing forces were exerted via pulling sites at confined positions around the indentation.

The end of the pre-invasive phase and the onset of BM transmigration is marked by a considerable change in the Fourier-filtered ERISM displacement pattern and the magnitude of substrate displacement. A total of 25.6 h after seeding the hd-BM acinus in Figure 4E transitioned from isolated, single pushing and pulling sites (Figure 4(Ev)) to a circular arrangement of multiple clearly distinguishable push/pull features with the pulling side facing the centre of the circle (Figure 4(Evi)). The latter is the characteristic fingerprint of isolated, single cells on an ERISM substrate that exert contractile forces via multiple FAs. Once the cells established stable adhesion contacts to the substrate, their surface areas and force increased further (Figure 4(Evii)).

As an extension to the earlier TFM analysis that demonstrated tangentially acting mechanical BM stresses, these data revealed that pre-invasive acini exert dynamic mechanical stress vertically at the BM–substrate interface, most probably due to actin-based, finger-shaped cell protrusions (see also Figure 3B–D). This BM stress was most prominent in a tumour microenvironment resembling conditions with the highest invasive outcome.

## 3. Discussion

### 3.1. Mechanical BM Integrity Is Essential to Counteract ECM Stiffness-Induced Cell Invasion

This study investigated the poorly understood interwoven response of breast gland acini to ECM stiffening, growth factor signalling and BM mechanics on the activation of BM stresses. Particularly, we draw a more comprehensive picture of BM-covered benign breast cells that underwent invasive transition. To investigate this transition from homeostatic to invasive breast tissue states in vitro, we used the commonly appreciated MCF10A acini model to study human breast gland morphogenesis [27]. Their BM thickness mimics those of mammary glands (104 nm to 1.4 µm) in vivo [28]. We previously showed that this experimental model enables growing acini either with thin (mean: 230 nm) and mechanically soft (0.35 kPa) ld-BMs, as well as with thick (mean: 660 nm) and thus mechanically stiffer (3.5 kPa) hd-BM scaffolds [18,19]. By tuning BM mechanics we studied the contribution of pathological BM thinning to irreversible BM disruption in nonmalignant breast gland tissue. Non-invasive BM thinning has been shown to regulate cell expansion in healthy mammary gland development [29].

In contrast, our results revealed a significant pro-invasive effect of mechanically impaired and thinned BM scaffolds. At the same time, this highlighted the relevance of BM-specific intact collagen IV meshwork as a cell invasion barrier. Further, we found that acinar cells sense substrate stiffening through their BM shielding responding with gradually increased invasive outgrowth. Supportive work showed a comparable ECM-stiffening effect in hydrogels with tuneable moduli [30]. We used elastomeric substrates with tuneable stiffnesses to resemble breast cancer-associated ECM stiffening found in vivo [23]. Our work contributes with the synergistic action of both tumour-like ECM stiffening and impaired BM mechanics facilitating BM invasion. We found that the transduction of ECM rigidity is mediated by acinar filopodia that pierce the BM scaffold without proteolytic-weakening. Breast acini use this mechanosensation mechanism in both physiological and pro-invasive environments.

### 3.2. Oncogenic EGF Signalling Overrides the Gradual Rigidity Response to Fuel BM Invasion

To resemble a tumour microenvironment, soluble EGF was applied as a paracrine oncogenic cue to activate EGFR downstream signalling. This further increased the already high invasiveness of acini induced by rigid substrates. Such a stiffness sensitizing effect has been demonstrated to foster EGF-induced contact-inhibited cell growth in 2D [31]. In addition, we now show that unleashed EGFR activation overrode the anti-invasive effect of healthy tissue compliance, which has not been reported for differentiated breast cell acini.

LD BM acini showed clear proliferative activity, which reflects their premature state [18]. After oncogenic EGFR activation, proliferation rates were further increased. This growth factor response fits the in vivo situation where pre-invasive breast cancer lesions could chronically stress their BM barrier by an expanding cell mass. The resulting expanding stresses could thus contribute to the increased BM invasiveness in our MCF-10A model.

In contrast, highly matured hd-BM acini had no such proliferative response to EGFR signalling but still reached a substantially induced invasive outcome. Hence, non-proliferative EGF signalling cascades have to favour the invasive transition. This is conceptually supported by the go or grow paradigm, whereby cancer cells are primed either for tumour growth or invasive spreading [32]. Our data indicate that oncogenic EGFR downstream signalling affects the invasive growth of breast gland acini either by triggering proliferation or motility depending on the current differentiation state of breast gland tissue.

### 3.3. Benign Breast Acini Activate MMP-Driven and Non-Proteolytic BM Disruption Modes

An important finding was that highly developed BM scaffolds asserted their primary invasion-blocking authority, even under the influence of invasion promoting growth factor signalling. We next aimed to distinguish the contribution of chemical and physical modes acinar cells could use to compromise their BM integrity. Thus, we mimicked excessive MMP proteolysis, as it is evident in vivo, and found potentiated invasiveness of beforehand low-invasive acini. This result confirmed the importance of an intact BM collagen IV meshwork to counteract local breast cell invasion. On the contrary, a blockade of cancer-linked soluble (MMP-1, -3, -7,-9) and membrane-bound (MMP-14) MMPs using a broadband MMP inhibitor [4,33] partially reduced BM disruption events in highly invasive breast acini. Admittedly, our BM invasion assay did not allow us to discriminate activated MMP classes or a potential contribution of serine-, aspartyl- and cysteinyl-proteases. However, the latter type has been linked to epithelial cancer [34] but not to BM degradation.

Together, our findings link invasion-specific collagenases to the BM-weakening process in invasion-triggered MCF10A breast acini. However, most interestingly, it also implicates the activation of an MMP-independent BM disruption mechanism. Oncogenic EGF and substrate stiffening resembled tumour-specific microenvironmental changes. Both drastically increased the invasiveness of breast acini. In turn, attenuation of oncogenic EGF signalling and MMP blockade together abolished the invasive phenotype. An ECM stiffness-induced activation of an MMP-14 and MMP-2/9 cascade has been shown for pancreatic cancer cells [35,36] and supports our new finding of ECM-triggered MMP activation in BM-covered breast acini. This further implies that MMP activity is mandatory for BM disruption under limited EGF conditions and triggered by tumour-associated substrate stiffening. In contrast to previous studies, our work revealed that tumorous ECM conditions activate the invasive transition of breast gland acini and local BM disruption without supporting MMP proteolysis. Non-malignant breast gland acini perform this non-proteolytic BM-weakening solely or together with proteolytic, depending on changing ECM conditions.

### 3.4. Invasion-Triggered Acini Generate Physical Stress to Chronically Weaken the BM Scaffold

Cell force-mediated BM breaching is essential for physiological processes and is vitally discussed as cancer cell invasion mode [5,6,7]. Naturally, all experimental BM invasion models are limited to reconstructing the in vivo situation of dynamically and heterogeneously developing entire tumour microenvironments. Nevertheless, our model system allowed, for the first time, mechanical BM stress quantification in dependency on changing tumour-specific ECM cues. We calculated the strain energy from the substrate deformation fields as a measure for the cell forces, which breast acini exerted on their underlying substrates through the BM barrier. A similar approach has been used to determine traction forces of contractile isolated breast cancer cells in a 3D collagen matrix [24].

Thereby, we explored 3D breast gland acini that exert forces acting on their own endogenous BM in a high temporal and spatial resolution that is currently technically impossible to realize in vivo. This approach revealed substantial force transmission through the BM scaffold onto the underlying substrate, thereby causing chronic BM stress that increased over several orders of magnitude until local BM breakdown. Our findings underline the importance of actin-based cell contractility for physical BM stress disruption that finally drives invasion. Thus far, reversible BM breaching has been demonstrated in mouse embryogenesis [37] and in *C. elegans* development [38].

In contrast to the latter work, our approach allowed us to quantify BM stress amplitudes depending on changing ECM conditions. Our finding that invasion-triggered breast acini exerted significantly increased force levels than under non-invasive conditions is important. Further, acini responded to tumour-like substrate stiffness with the highest force amplitudes that are prone to chronically weaken the BM barrier. Such modulation of BM stress has not been reported for breast acini so far. This implicates a signalling-loop between E substrate stiffening and physical BM stress caused by constrained cell motion within the BM shell. Actomyosin-dependent cell motion is well-regulated under physiological microenvironmental conditions and essential for BM maintenance and homeostasis [39]. In contrast, we show that tumour ECM-induced cell contractility could thus lead to chronically imbalanced solid shear strain, as well as and tensile stress at the BM–matrix interface.

Further, EGF-activated ld-BM acini showed the most substantial force amplitudes. These acini also exhibited an increased fraction of dividing cells. Dividing cells expand along their mitotic axes and generate additional compressive forces towards the BM [40]. Accordingly, the observed low invasiveness of low-proliferative and hence nonexpansive hd-BM acini could relate to lower BM stress levels. Moreover, matured hd-BMs are substantially thicker than ld-BMs and have significant resistance against compression (800 Pa) [18]. Matured BM mechanics could hence account for the high degree of stress compensation we found for hd-BM acini. Notably, the non-invasive breast acini fractions that lacked BM disruption generally showed substantially lower BM stress levels even when stimulated with tumour-like conditions. This finding suggests that certain stress thresholds BMs withstand before losing structural integrity.

Together our TFM data show that benign breast acini use trans-BM mechanosensation and (non)proliferative EGF signalling cascades to activate an efficient force-driven mode of BM disruption mediated by cellular expansion and contractility. These imbalanced physical stresses are counteracted only by the given mechanical BM strength, which can be further compromised by tumour ECM-induced MMP proteolysis.

### 3.5. Mechanosensitive Filopodia Contribute to Physical BM Stress and Invasion

As a cellular mechanism of trans-BM ECM mechanosensation, we discovered the formation of mechanically active F-actin-based filopodia that squeeze through the BM collagen IV network without the need for proteolytic widening. The mechanotransducer FA protein vinculin [41,42] colocalized with the tips of BM traversing F-actin microspikes and indicated the formation of force-transmitting FA complexes. Further, the periodic oscillation time (4.0 min) matched those described for FA-stabilized (5.7 min) [43] and force-transmitting filopodia [44]. An intriguing finding was the protrusive substrate deformation pattern perpendicular to the BM–substrate interface. These tightly confined active pulling and pushing patterns were present underneath the acinar body directly after substrate adhesion that continuously increased in the invasion process and peaked at timepoints of BM breakdown. These findings showed that pre-invasive breast acini form thin filopodia, which transverse the collagen IV meshwork of their intact BM to adhere and probe ECM mechanics. In pro-invasive stiff ECM conditions, these mechanosensitive filopodia transmitted dynamic push and pull forces to the ECM and consequently to the BM. Here, early stress onset and the largest stress amplitudes correlated with the highest invasive outcome. Hence, those protrusive forces could contribute to chronic physical BM stress and contractility-based tangential BM stress in invasion-triggered breast acini. It is noteworthy that the high amplitude of stress we measured for individual filopodia has been described, so far, instead of matured invadopodia in head and neck cancer cells [45]. Hence, acinar filopodia are likely to convert into finger-like proteolytically active invadopodia, as shown for other cancer cells [46]. EGFR signalling regulates such functional conversion [47] and could explain the extensive BM proteolysis activation in our EGF-stimulated breast acini model. Additionally, non-proteolytic filopodia have been accounted for BM breaching in MMP deficient mice as well [48]. Our data do not rule out the latter mechanism, but the reductive effect of MMP inhibition argues for the presence of MMP-releasing invadopodia, at least in later stages of BM-weakening. To further link the newly found BM stress generating filopodia to the BM invasion process, we inhibited PI3-Kinase activity to short circuit a central signalling hub for filopodia/invadopodia formation, MMP activation [49] and ECM mechanotransduction [30,50]. Thus far, the contribution of PI3-K activity to BM disruption in breast acini remained elusive. Our work confirmed the substantial modulation of BM invasion by PI3-K downstream signalling. Since PI3-K inhibition resulted in a complete invasion block, it is most reasonable that it modulates both chemical and physical BM-weakening in benign breast cells. Of note, our findings do not allow a detailed description of the underlying signalling circuits and future endeavours should address the associated mechanotransductive circuits.

## 4. Materials and Methods

### 4.1. Cell Maintenance

MCF10A cells were purchased from ATCC (Manassas, VA, USA) and maintained in standard culture conditions (37 °C, 5% CO_2_) in DMEM/F12 (ThermoFisher Scientific, Waltham, MA, USA) containing 5% horse serum (ThermoFisher Scientific, Waltham, MA, USA), 0.5 µg/mL hydrocortisone, 100 ng/mL cholera toxin, 20 ng/mL EGF, 10 µg/mL insulin, 100 U/mL penicillin and 100 µg/mL streptomycin (Sigma Aldrich, Darmstadt, Germany).

### 4.2. MCF10A Morphogenesis and Basement Membrane Invasion Assay

Single MCF10A cells were seeded on top of growth factor reduced EHS substrate (Geltrex, ThermoFisher Scientific, Waltham, MA, USA) and to generate ld-BM (day 10) and hd-BM (day 21) acini and gently isolated from EHS matrix as described in [18] with slight modifications: EHS-bound acini were gently washed 5 min with ice-cold PBS and incubated in 2 mL ice-cold Cell Recovery Solution (CRS) (BD Biosciences, San Jose, CA, USA) for 30 min (4 °C) to depolymerize the EHS matrix and were replaced by EGF-free medium. Individual acini were picked under a stereomicroscope, washed with fresh EGF-free medium and seeded onto TFM and ERISM substrates. Acini were let to adhere for 10 min (37 °C, 5% CO_2_) and covered with 4 mL medium. The seeding timepoint was defined as assay start.

### 4.3. Elastomeric Substrate Preparations and EHS Protein Coating

Cells were seeded on cross-linked silicone rubber substrates with varying elasticity (Sylgard 184, Dow Corning, Midland, MI, USA). Mixing ratios of 50:1 and 73:1 (m/m) for base oil and cross-linker oil produced 12 and 0.12 kPa substrates, respectively. For invasion assays, elastomers were spin coated as 70 μm thin layers over 110μm thin cover slides (Cover Slip, Ø18 mm, #0, Menzel-Gläser, Braunschweig, Germany) and glued under a hole drilled into a Petri dish [51]. Characterization of elastomer material properties (Young’s modulus and Poisson’s ratio) was performed as described previously [52]. For cell seeding, substrates were coated with a thin, non-gelling protein solution of Geltrex (20 µg/mL, in ice-cold PBS, overnight at 4 °C).

### 4.4. Proliferation Assay

Cell proliferation within individual breast acini was measured by detecting newly formed nuclei (Click-iT AlexaFluor 488 Imaging Kit, ThermoFisher Scientific, Waltham, MA, USA). In brief, acini were incubated with 10 µM of 5-Ethynyl-2′-deoxyuridine (EdU) for 30 h and newly synthesized DNA (EdU positive) was coupled with Alexa488 fluorophore. Acini were fixed and counter stained with NucBlue (R37606, ThermoFisher Scientific, Waltham, MA, USA) to detect the total number of nuclei. Confocal image stacks of whole acini were recorded. IMARIS 9.1 software (Bitplane, Zürich, Switzerland) was used for semiautomatic nuclei counting within individual samples by detecting the fluorescence signals for NucBlue positive and (EdU and NucBlue double positive nuclei). The cell proliferation index was calculated as a ratio of these two nuclei fraction.

### 4.5. Biochemical Treatments

After EHS isolation and seeding onto substrates acini were incubated either with EGF-free medium or with medium containing 20 mg/mL EGF [18]. For BM manipulation, acini were treated for 30 min with collagenase IV (290 U/mL, Worthington Biochemical Corp., Lakewood, NJ, USA) [18]. For MMP inhibition 20 µM Marimastat (BB-2516) was added for 65 h. For PI3-K inhibition acini were treated with low dose of (25 nM) Wortmannin (KY 12420) in medium (Sigma-Aldrich, Darmstadt, Germany) for one hour.

### 4.6. Microscopy

Live cell imaging was carried out at 37 °C and 5% CO_2_ (cell incubator XL, Zeiss, Jena, Germany) using an inverted microscope (Axiovert 200, Zeiss, Jena Germany), equipped with a AxioCam MRm camera (Carl Zeiss, Jena, Germany) and an EC Plan-Neofluar 40× oil immersion objective (PH3, NA 1.30, Zeiss, Germany). Images were taken using the ZEN 2.3 blue edition software (Zeiss, Jena, Germany). Fluorescent beads were excited with a mercury arc lamp (HXP-120, Osram, Munich, Germany). The filter set 64 HE (Zeiss, Jena, Germany) was used. Multiposition and tile scan (field of analysis: 459.3 × 614.9 µm) experiments allowed us to analyse several acini simultaneously. Immunofluorescently labelled fixed cells were analysed with inverse confocal laser scanning microscopes (LSM710 and LSM880 with an Airyscan detector, Zeiss, Jena, Germany) using a 40× Plan-Apochromat water immersion objective (NA 1.1, Zeiss, Germany). Confocal micrographs were taken using an argon ion laser (488 nm) and helium–neon laser lines (543 and 633 nm) combined with the appropriate emission bandpass filters.

### 4.7. Traction Force Microscopy

Substrate deformations caused by traction forces were visualized by tracking fluorescent beads that were immobilized on top of elastomeric substrates, as described elsewhere [53]. The image recording interval was 20 min over 65 h. Maps of cell-induced traction stresses were calculated by regularized least square fitting to the mechanical response of an elastic layer of 80 µm thickness on rigid substrates [21]. From these maps, strain energy was calculated as scalar measure of overall mechanical activity of cells (for detailed information see caption of Figure S1). The strain energy calculation was based on previous work on TFM techniques [21,25,54,55]. Algorithms were implemented in MatLab (R2015a, The MathWorks Inc., Natick, MA USA). The fold change (FC) of strain energy between two sample groups was calculated as ratio of mean values for each timepoint. The FC_mean_ is the average of the FC of all timepoints.

### 4.8. Elastomer Resonance Interference Spectroscopy Microscopy (ERISM)

The fabrication of ERISM substrates and their readouts is described in detail elsewhere [22]. In brief, ERISM substrates consist of an 8 µm thick layer of a soft elastomer situated between two semitransparent, mechanically flexible gold mirrors which form an optical microcavity with an apparent stiffness of 3 kPa. Mechanical force exerted by the acinus caused local deformations of the microcavity and thus local shifts of its resonance wavelengths. The resulting interference patterns were recorded by monochromatic imaging of the microcavity reflectance at different wavelengths and analysed by optical modelling in order to compute a high-resolution displacement map with µm lateral resolution and nm displacement resolution. FEM conversion into stress maps allowed for the detection of forces in the pN range.

### 4.9. Immunofluorescence Techniques

EHS embedded acini were fixated with 2% formaldehyde (Sigma-Aldrich) and 0.5% glutaraldehyde (EM Grade, Polysciences, Inc., Warrington, PA, USA) and stained as described previously [18]. Transferred acini were fixed using 3.7% formaldehyde in cytoskeleton-buffer (CB: 5 mM EGTA, 5 mM glucose, 10 mM MES, 5 mM MgCl_2_, 150 mM NaCl, 1 g/L streptomycin; Sigma Aldrich, Darmstadt, Germany) for 20 min at room temperature (RT). Samples were washed (2 × 5 min) with 30 mM glycine-CB at RT, permeabilized with 0.5% Triton X100-CB (Sigma-Aldrich, Darmstadt, Germany) solution for 20 min and treated with blocking solution (5% skim milk powder (Sigma-Aldrich) and 1% AffiniPure F(ab’)_2_ fragment goat antimouse IgG, 115-006-006, Jackson ImmunoResearch, West Grove, PA, USA) in CB for 2 h at RT. Primary antibodies (antitype IV collagen ab6586, Abcam, Cambridge, England, UK; antivinculin clone hVin-1, V9131 Sigma-Aldrich, Darmstadt, Germany) were diluted in 1% skim milk powder in CB and incubated overnight at 4 °C. Secondary antibodies conjugated with fluorescent dyes (ThermoFisher Scientific, Waltham, MA, USA) were diluted (1:200) in 1% skim milk powder in CB and applied for 45 min. (RT, darkness). Alexa Fluor 488/546/633 Phalloidins (ThermoFisher Scientific, Waltham, MA, USA) were applied simultaneously. Nuclei were counterstained either with DRAQ5 (4084, Cell Signaling, Danvers, MA, USA) or NucBlue (R37606, ThermoFisher Scientific, Waltham, MA, USA) for 10 min (RT).

### 4.10. Statistical Analyses

Groups were compared using the two-sided Chi-square test with Yates’ correction for trend (Figure 1E,G,L), the two-tailed nonparametric Mann–Whitney test (95% confidence interval) (Figure 2E–G), the One-Way ANOVA followed by Tukey’s multiple comparisons test (Figure 1H; Figure 2E,F) and the two-tailed Student’s *t*-test (Figure 4G,H). Statistical tests were performed using GraphPad Prism version 7.05 (GraphPad Software, La Jolla, CA, USA) and OriginPro, Version 2019 (OriginLab Corporation, Northhampton, MA, USA). *p*-values were defined as follows: n.s.: *p* ≥ 0.05; *: *p* < 0.05; **: *p* < 0.01; ***: *p* < 0.001; ****: *p* < 0.0001).

## 5. Conclusions

**Figure 5:**
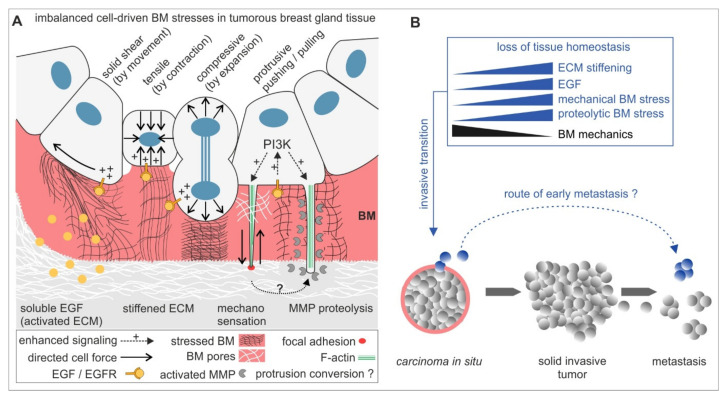
A mechanistic model for BM disruption and invasive dissemination in benign breast gland tissue. (**A**) The mechano-response of non-invasive breast acini to tumour promoting ECM cues activating mechanical and proteolytic stress modes. These modes promote BM-weakening to facilitate invasion synergistically or solely. In the early process of invasive transition, BM mechanics are decisive for stress compensation and to attenuate cell invasion. (**B**) The BM fails to withstand these cellular stresses in progressive tumour microenvironments that induce tissue homeostasis loss. A fraction of benign cells undergoes an invasive transition to transmigrate the locally weakened BM. Finally, this enables a very early tumour cell dissemination and metastasis mode that skips an in situ progression with solid tumour mass expansion and local tissue infiltration.

The present study led to an extended concept of biomechanically regulated BM invasion (see Figure 5): chemical and mechanical tissue parameters synergistically activate the invasive transition of originally non-malignant breast gland acini. This tissue response steers intra-acinar motion, contractility and thus high cellular forces and mechanically stressing protrusions, which together weaken the BM barrier chronically and result in an imbalanced, non-homeostatic breast cell cluster. BM mechanics compensate these stresses partially to attenuate the invasive transition. MMP proteolysis accelerates BM-weakening, most-likely by PI3-K-dependent invadopodia maturation. Breast cancer progression is characterized by a stepwise growth from non-invasive lesions to locally expanding and tissue infiltrating tumour masses from which eventually invasive tumour cells disseminate to metastasize. In addition to this, our new results emphasize the activation of an alternative route of metastasis that grounds on very early cell dissemination events from pathologically non-invasive BM-covered *carcinoma* in situ (*CIS).* In line with this, CTSs have been found in breast cancer patients suffering from non-invasive *DCIS* [11]. Our force-driven local BM disruption model supports this controversial paradigm of metastasizing CTCs patients with *CIS* lesions. However, it remains desirable to translate our findings into a model that integrates pre-invasive breast tissue lesions and tumour tissue-related cells, i.e., cancer-associated fibroblasts and macrophages, to explore the paracrine contributions to early BM disruption in *CIS*. Finally, this is precious knowledge that could hopefully be applied to prevent the transition of pre-invasive breast lesions to invasive carcinomas.

## Data Availability

The datasets supporting the conclusions of this article are either available within the paper and its supplementary information files or from the corresponding author upon reasonable request.

## Supplementary Materials

The following are available online at https://www.mdpi.com/article/10.3390/ijms22083962/s1, Figure S1: Noise floor of strain energies, Figure S2: Proliferation of acinar cells during BM-invasion, Figure S3: Basement membrane traversing cell protrusions, Figure S4: Temporal evolution of the substrate volume displaced by the mechanical activity, Figure S5: Comparison of the mean indented volume during pre-BM transmigration mechanical activity of ld-BM and hd-BM acini; Table S1: Exact invasive fraction values and mean invasion timepoints, Table S2: Exact median strain energy values, Movie S1: Time-lapse sequence over 65 h of the ld-BM acinus on tumour-like (12 kPa) stiffness conditions, shown in Figure 2C,E,G, Movie S2: Time-lapse sequence over 65 h of the hd-BM acinus on normal-like (0.12 kPa) stiffness conditions, shown in Figure 2F,G, Movie S3: ERISM time-lapse investigation of the ld-BM acinus shown in Figure 4E, Movie S4: ERISM time-lapse investigation of the hd-BM acinus shown in Figure 4H, Movie S5: ERISM time-lapse investigation of the ld-BM acinus discussed in Figure 4K.
