# Supplementary material for: Cell Force-Driven Basement Membrane Disruption Fuels EGF- and Stiffness-Induced Invasive Cell Dissemination from Benign Breast Gland Acini
Source: Int J Mol Sci. 2021 Apr 12;22(8):3962. doi: 10.3390/ijms22083962 (PMC8070162; doi:10.3390/ijms22083962)
Supplement: Supplementary file 1 [file ijms-22-03962-s001.zip › ijms-1161653 supplementary.v3_proof.pdf]

Supplementary Material

Cell Force-Driven Basement Membrane Disruption Fuels EGF- and Stiffness-Induced Invasive Cell Dissemination from Benign Breast Gland Acini

Aljona Gaiko-Shcherbak [1,†], Julian Eschenbruch [1,†], Nils M. Kronenberg [2,3], Michael Teske [1], Benjamin Wolters [1], Ronald Springer [1], Malte C. Gather [2,3], Rudolf Merkel [1], Bernd Hoffmann [1] and Erik Noetzel [1,*]

[1] Institute of Biological Information Processing 2 (IBI-2): Mechanobiology, Forschungszentrum Jülich, 52425 Jülich, Germany; agaiko-shcherbak@clinipace.com (A.G.-S.); j.eschenbruch@fz-juelich.de (J.E.); michael.teske@uzh.ch (M.T.); benjamin.wolters@evotec.com (B.W.); r.springer@fz-juelich.de (R.S.); r.merkel@fz-juelich.de (R.M.); b.hoffmann@fz-juelich.de (B.H.)

[2] SUPA, School of Physics and Astronomy, University of St Andrews, St Andrews KY16 9SS, UK; nils.kronenberg@uni-koeln.de (N.M.K.); mcg6@st-andrews.ac.uk (M.C.G.)

[3] Humboldt Centre for Nano- and Biophotonics, Department of Chemistry, Universität zu Köln, 50939 Köln, Germany

* Correspondence: e.noetzel-reiss@fz-juelich.de

† These authors share the first authorship.

Movie S1:

Time-lapse sequence over 65 hours of the ld-BM acinus on tumour-like (12 kPa) stiffness conditions, shown in Fig 2C, E and G.

Movie S2.

Time-lapse sequence over 65 hours of the hd-BM acinus on normal-like (0.12 kPa) stiffness conditions, shown in Fig 2F and G.

Movie S3.

ERISM time-lapse investigation of the ld-BM acinus shown in Fig 4E. Phase contrast microscopy (left), substrate displacement in nm (middle) and spatial Fourier-filtered substrate displacement (right).

Movie S4.

ERISM time-lapse investigation of the hd-BM acinus shown in Fig 4H. Phase contrast microscopy (left), substrate displacement in nm (middle) and spatial Fourier-filtered substrate displacement (right).

Movie S5.

ERISM time-lapse investigation of the ld-BM acinus discussed in Fig 4K. Phase contrast microscopy (left) and spatial Fourier-filtered substrate FEM stress in Pa (right) showing the inset marked in the phase contrast image.

A

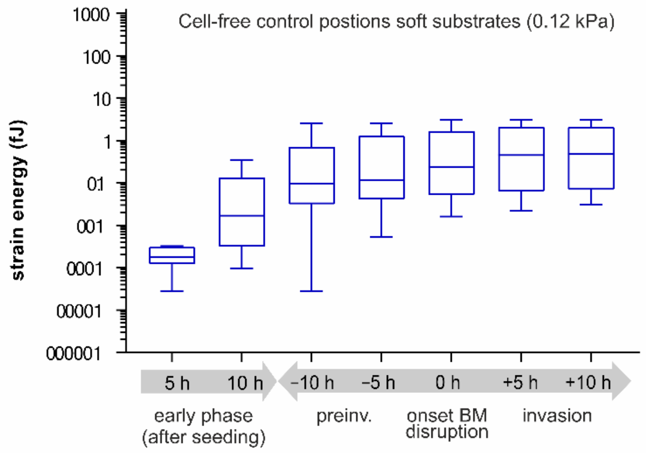

	5 h	10 h	−10 h	−5 h	0 h	+5 h	+10 h
Number of values	6	7	80	81	81	81	74
Minimum	0.0002765	0.0009434	0.0002765	0.005279	0.01606	0.02171	0.0308
25% Percentile	0.001214	0.003146	0.03124	0.04112	0.05163	0.06341	0.07082
Median	0.001765	0.0168	0.09674	0.1164	0.236	0.456	0.4825
75% Percentile	0.003092	0.1353	0.7183	1.3	1.689	2.137	2.094
Maximum	0.003196	0.3432	2.529	2.543	3.138	3.16	3.16

B

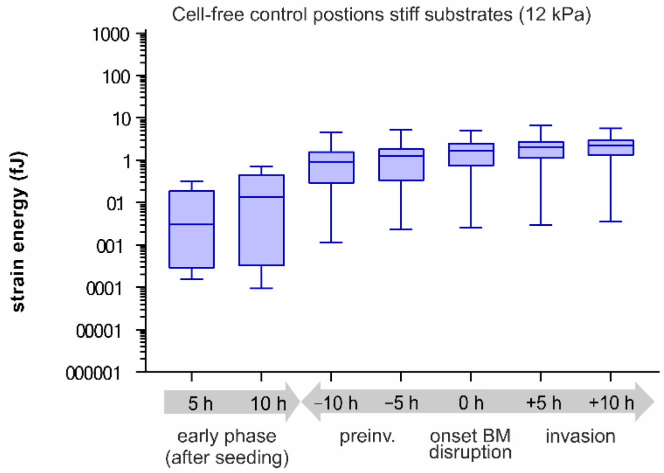

	5 h	10 h	−10 h	−5 h	0 h	+5 h	+10 h
Number of values	6	7	74	79	83	82	75
Minimum	0.001564	0.0009434	0.0117	0.0232	0.02596	0.02955	0.03587
25% Percentile	0.002788	0.003146	0.281	0.3233	0.7158	1.076	1.277
Median	0.03027	0.1353	0.9237	1.274	1.655	2.072	2.27
75% Percentile	0.195	0.4725	1.638	1.914	2.608	2.878	3.141
Maximum	0.3233	0.7158	4.459	5.229	5.002	6.566	5.703

Figure S1. Noise floor of strain energies. Overall comparison of strain energy values recorded for cell-free control positions. One control position was measured for each independent experiment. *(A)* The unspecific measurement noise for soft substrates and *(B)* the according values for stiff ones. X-axis: continuous data, normalized for each sample by defining the individual *onset of BM transmigration* as hour zero (0 h) and dichotomized into *pre-* (-h) and *post-* (+h) *invasive phases* (hours). Early phase: first 5 and 10 hours after seeding (= assay start).

Scatter bars: median with 95% CI. The calculations of strain energy from the stress maps of cell-free substrates and substrates populated with MCF10A acini are based on previous work on TFM techniques [21, 25, 54, 55]. Strain energy is used as a robust and scalar measure for cellular traction force [24].

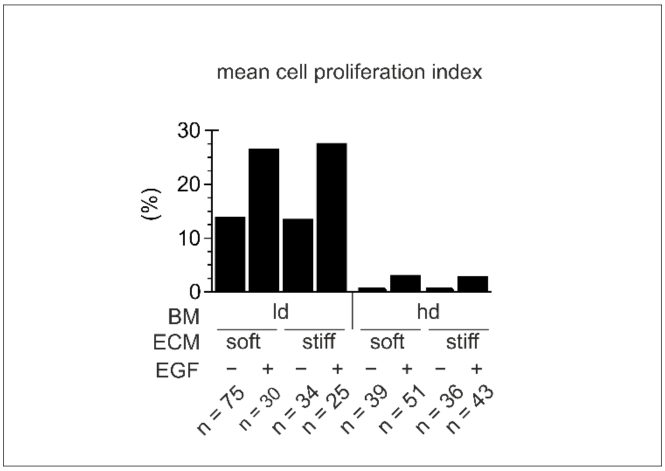

Figure S2. Proliferation of acinar cells during BM-invasion. Cell proliferation was measured in acini groups that were applied to the BM invasion assay after 30 hours of incubation. 30 hours were chosen as readout since this is in the range of mean BM invasion onset of the most invasive ld-BM breast acini groups (see also Fig 1E) The histogram highlights the mean proliferative nuclei fractions within each acini group, in absence and presence of EGF (20 ng/mL), as well as dependent of normal- and tumour-like tissue states. n: number of analyzed acini from at least three independent experiments.

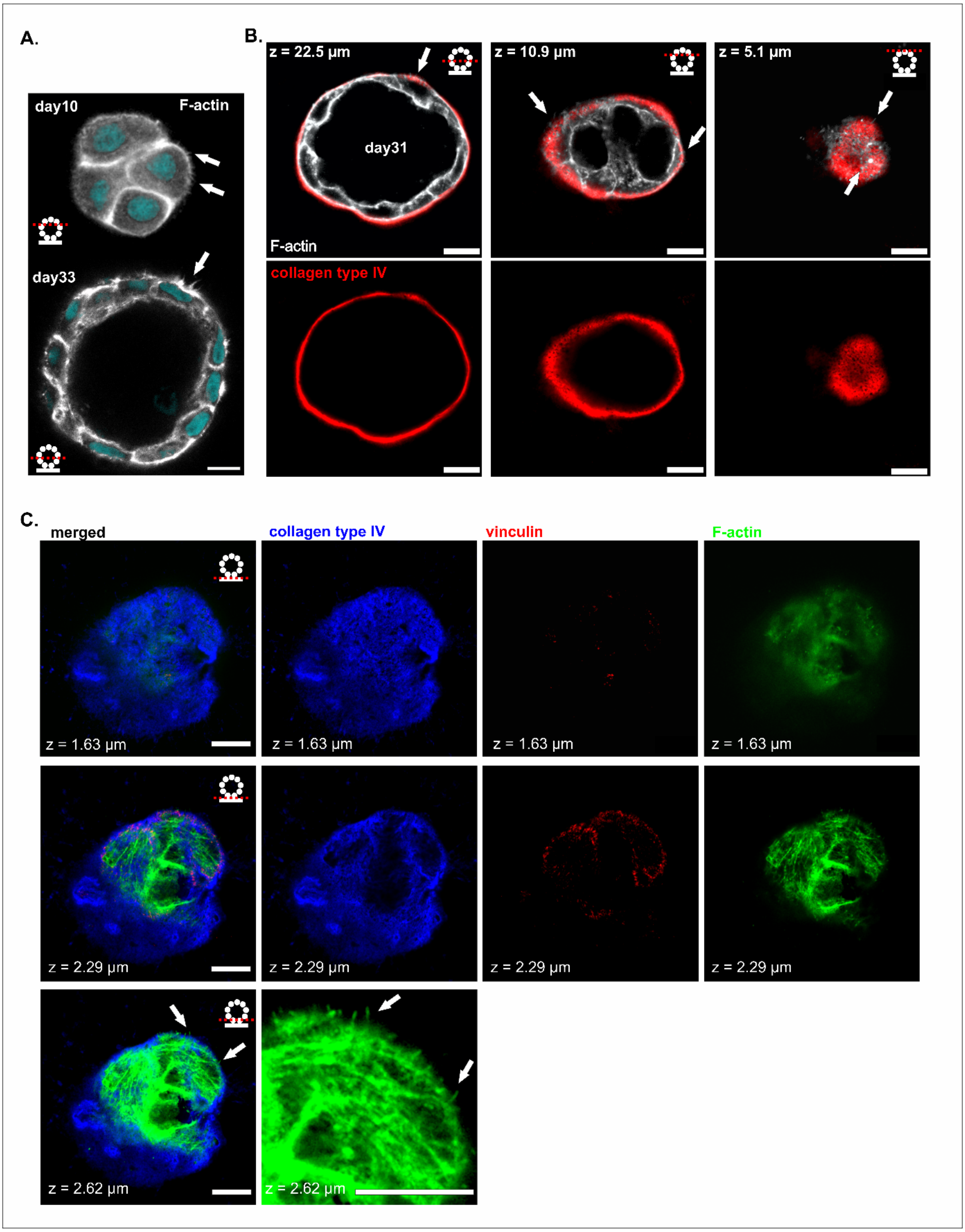

Figure S3. Basement membrane traversing cell protrusions. Hd-BM acini were fixed and IF stained. *(A)* Confocal cross sections show the presence of randomly formed actin-based protrusions (F-actin, grey; arrow heads) formed in physiological conditions by maturing ld-BM acini (day 11) and highly-developed, resting hd-BM acini after 33 days in EHS matrix. Cyan: nuclei. *(B)* Image series at different z-planes of a resting hd-BM acinus (day 31) to illustrate protrusion bundles (arrow heads) oriented towards the extracellular space (cf, Fig 3A). Actin

spikes and fingers traverse through the intact BM-scaffold (red). *(C)* Image series at different z-planes shows the acini-BM-substrate contact plane of an hd-BM acinus, 48 hours after seeding on a glass coverslip. Detailed view on additional image z-planes proximate to the BM-substrate interface (cf, Fig 3C and D) demonstrates the distribution lateral distribution of actin-rich protrusions and the co localization of F-actin spots (green) and vinculin spots (red) within collagen type IV pores (blue) before BM transmigration. Scale bars: 10 μm.

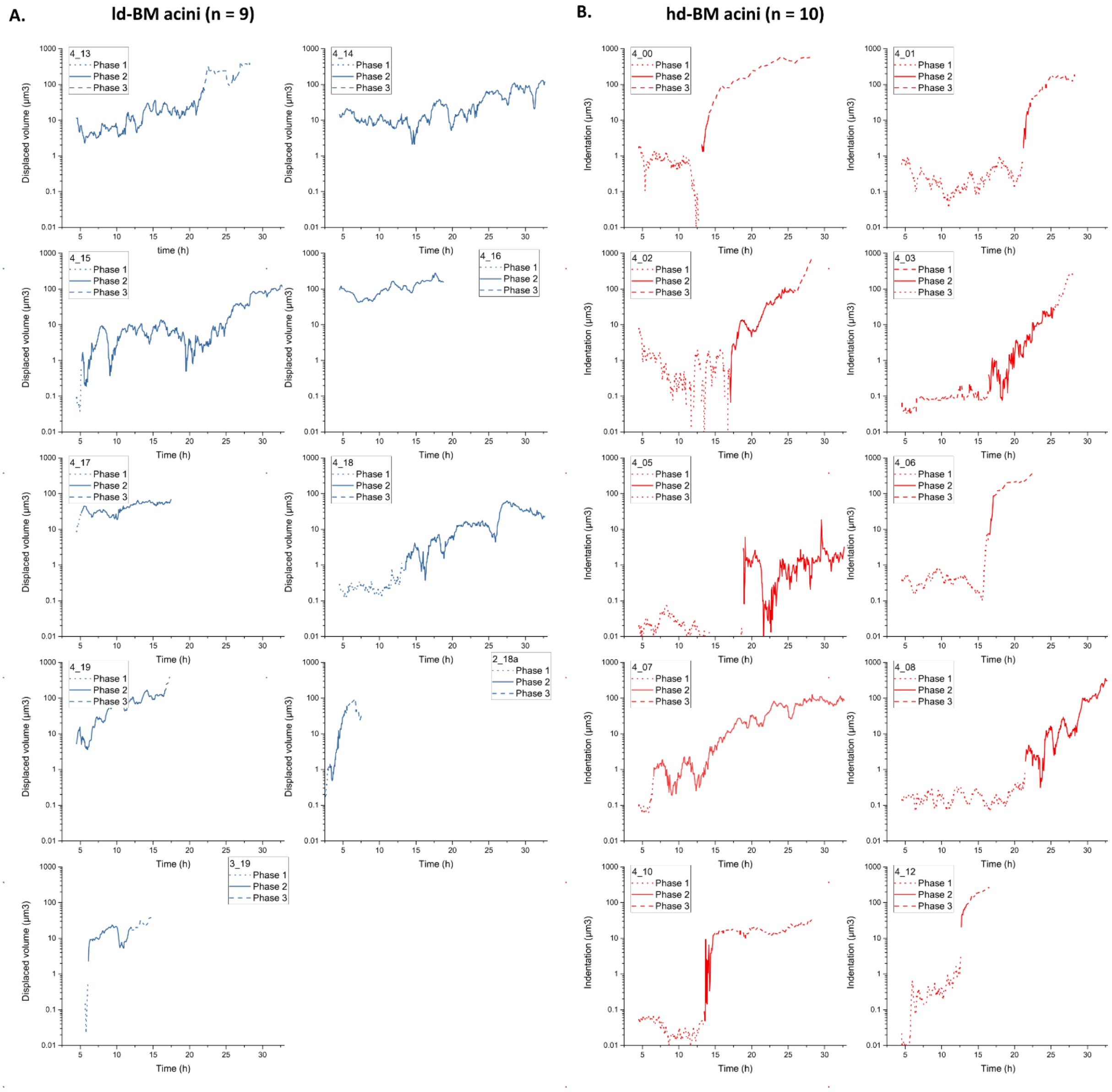

Figure S4. Temporal evolution of the substrate volume displaced by the mechanical activity of *(A)* all investigated ld-BM acini and *(B)* all investigated hd-BM acini. Dotted lines indicate the phase before pre-BM-transmigration mechanical activity. Solid lines show pre-BM-transmigration mechanical activity. Dashed lines show mechanical activity of isolated, single cells after successful BM-transmigration.

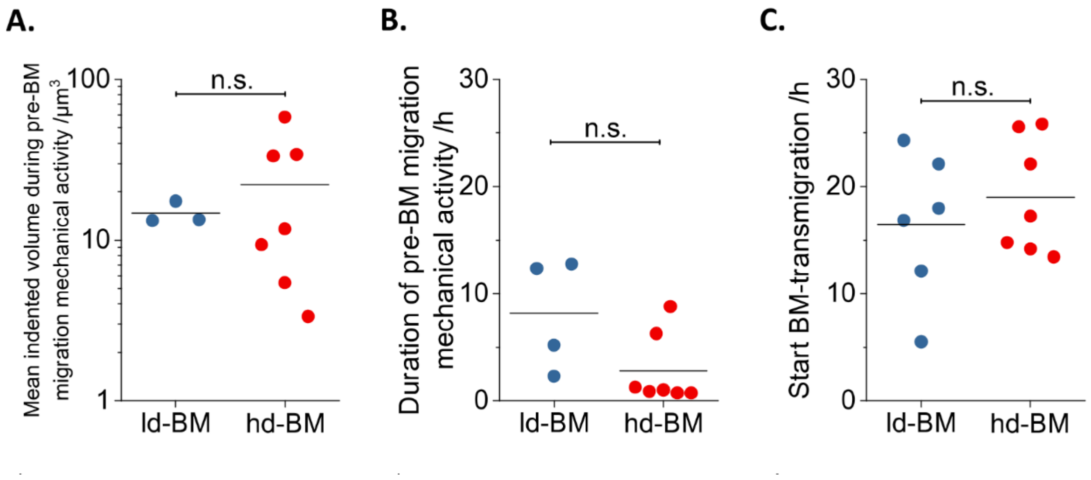

Figure S5. *(A)* Comparison of the mean indented volume during pre-BM transmigration mechanical activity of ld-BM and hd-BM acini. Ld-BM acini (blue; n = 3) and hd-BM acini (red; n = 7). *(B)* Comparison of the duration of pre-BM-transmigration mechanical activity of ld-BM (blue; n = 4) and hd-BM (red; n = 7) acini after seeding the acini on ERISM chip. Groups were compared using Student's *t*-test; n.s.: $p > 0.05$.

Table S1. Exact invasive fraction values and mean invasion timepoints. Basement membrane mechanics counteract tumour ECM-induced invasion. This table shown the values that are displayed for the invasive acini fractions (%) and the individual invasion onset timepoints (hours) in the manuscript Figure 1E, G, H, I, J, L and M. Confidence interval (CI); stiff ECM = 12 kPa substrate, soft ECM = 0.12 kPa substrate

	**without EGF**				
	**hd-BM / soft ECM**	**hd-BM / stiff ECM**	**ld-BM / soft ECM**	**ld-BM / stiff ECM**	
**Fig 1E**					
invasive acini fraction	2%	10%	20%	28%	
	**20ng/mL EGF**				
	**hd-BM / soft ECM**	**hd-BM / stiff ECM**	**ld-BM / soft ECM**	**ld-BM / stiff ECM**	
**Fig 1G**					
invasive acini fraction	53%	59%	82%	80%	
**Fig 1H**					
mean (h)	43.05	42.28	32.2	30.85	
lower CI (h)	39.50	39.10	28.90	26.48	
upper CI (h)	46.60	45.47	35.50	35.21	
**Fig 1I + collagenase**					
cum. onset of invasion	100%				
**Fig 1J + collagenase**					
mean (h)	28.43				
lower CI (h)	25.28				
upper CI (h)	31.58				
					**without EGF**
**Fig 1L +marimastat**					**ld-BM / stiff ECM**
invasive acini fraction	23%	32%	51%	44%	5%
**Fig 1M +marimastat**					
mean (h)	46.92	45.65	37.89	38.11	
lower CI (h)	42.31	41.01	33.38	34.69	
upper CI (h)	51.53	50.30	42.41	41.53	

Table S2. Exact median strain energy values. Mechanical BM-stress exertion by benign breast acini. This table shown the exact strain energy values (in femtoJoule, fJ) that are displayed for the invasive acini fractions in the manuscript Figure 2E and F. Confidence interval = CI. Timepoints: the first 5 hours and 10 hours after acini seeding define the early phase. Other timepoints are normalized to the individual onset of BM disruption (= 0 h) and dichotomized into comparable pre- ("- hours") and postinvasive ("+ hours") phases; stiff ECM = 12 kPa substrate, soft ECM = 0.12 kPa substrate

	**ld-BM / soft ECM**						
**timepoints [h]**	**5**	**10**	**−10**	**−5**	**0**	**+5**	**+10**
median (fJ)	0.0086	0.41	1.18	1.69	4.65	11.59	24.66
lower CI (fJ)	0.0052	0.22	0.7671	1.16	1.40	4.75	7.89
upper CI (fJ)	0.014	0.97	2.56	3.03	8.05	16.19	42.07
	**ld-BM / stiff ECM**						
**timepoints [h]**	**5**	**10**	**−10**	**−5**	**0**	**+5**	**+10**
median (fJ)	7.47	33.01	34.83	36.7	50.48	78.47	104.5
lower CI (fJ)	1.04	16.22	21.57	24.63	33.71	50.85	63.53
upper CI (fJ)	21.87	59.47	56.98	54.48	80.79	124.5	154.4
**Fig 2F**	**hd-BM / soft ECM**						
**timepoints [h]**	**5**	**10**	**−10**	**−5**	**0**	**+5**	**+10**
median (fJ)	0.028	0.173	2.43	3.25	3.24	11.08	27
lower CI (fJ)	0.0075	0.072	1.27	1.46	2.19	6.13	14.58
upper CI (fJ)	0.068	0.54	3.52	5.07	7.05	30.96	62.31
	**hd-BM / stiff ECM**						
**timepoints [h]**	**5**	**10**	**−10**	**−5**	**0**	**+5**	**+10**
median (fJ)	0.13	4.93	20.2	25.94	33.36	64.91	111.3
lower CI (fJ)	0.09	2.83	12.09	19.72	29.39	45.01	82.74
upper CI (fJ)	0.22	8.41	31.03	34.42	49.81	102.2	137.6
